# Supplementary material for: Design and fabrication of flexible DNA polymer cocoons to encapsulate live cells
Source: Nat Commun. 2019 Jul 3;10:2946. doi: 10.1038/s41467-019-10845-2 (PMC6610073; doi:10.1038/s41467-019-10845-2)
Supplement: Supplementary file 1 — Supplementary Information [file 41467_2019_10845_MOESM1_ESM.pdf]

Supplementary Information for

Design and fabrication of flexible DNA polymer cocoons to encapsulate live cells

Gao et. al.

Supplementary Tables:

Supplementary Table 1. Optimization of the reaction buffer and the enzyme used for isDOP. The efficiency of reactions is evaluated by the amount of generated DNA polymers. Buf. 3 and Klenow DNA polymerase are tested in the experiments. Data are presented as the mean ± standard division (s.d.) of three independent experiments.

Buffers	Components	Enzymes	Reaction Efficiency (%)
H_2O	—	phi 29	0
		Klenow	0
Buf. $_{phi29}$	50 mM Tris, 10 mM $MgCl_2$, 10 mM $(NH_4)_2SO_4$, 4 mM DTT, pH 7.5 @ 25°C	phi 29	95±5
		Klenow	—
Buf. $_{klenow}$	10 mM Tris, 10 mM $MgCl_2$, 50 mM NaCl, 1 mM DTT, pH 7.9 @ 25°C	phi 29	—
		Klenow	100±3
Buf. 1	DMEM (25 mM HEPES)	phi 29	2±1
		Klenow	7±2
Buf. 2	DMEM, 10 mM $MgCl_2$, 10 mM $(NH_4)_2SO_4$, 4 mM DTT, pH 7.2 @ 25°C	phi 29	5±1
		Klenow	75±3
Buf. 3	DMEM, 10 mM $MgCl_2$, 1 mM DTT, pH 7.2 @ 25°C	phi 29	5±1
		Klenow	86±6

Supplementary Table 2. DNA sequences employed in this study. The nucleotides in color represent the encoding sequences (ES_1, ES_2 and ES_3), and the underlined nucleotides represent the recognition regions of high-fidelity restriction endonucleases (EcoRI, HindIII, and PstI, respectively). The encoding sequences are designed to encode cells and also to introduce new interfaces for cell manipulation.

Name	Sequence
**IP_1**	5' - **DSPE-PEG$_{2000}$** - TTTTTTTTTT**AGACACTATATGACA** - 3'
**IP_2**	5' - **SDA** - TTTTTTTTTT**AGACACTATATGACA** - 3'
**F-IP_1**	5' - **DSPE-PEG$_{2000}$** - TTTTTTTTTTAGACACTATATGACA - **FAM** - 3'
**F-IP_2**	5' - **SDA** - TTTTTTTTTTAGACACTATATGACA - **FAM** - 3'
**BP**	5' - **AATGTCAACGCA** - 3'
**BP'**	5' - **AATGTCAACGCA** - **FAM** -3'
**cirDNA$_1$**	5' - TCAACCCTCTCGTTT**CTTGAATTCCTG**(**ES$_1$**)TTATTTTCATA CAATGTCAACGCAC**TGTCATATAGTGTCT**CCTGAATGTCCTAT CC - 3'
**cirDNA$_2$**	5' - TCAACCCTCTCGTTT**CTTAAGCTTCTG**(**ES$_2$**)TTATTTTCATA CAATGTCAACGCACTGTCATATAGTGTCTCCTGAATGTCCTATC C - 3'
**cirDNA$_3$**	5' - TCAACCCTCTCGTTT**CTTCTGCAGCTG**(**ES$_3$**)TTATTTTCAT ACAATGTCAACGCACTGTCATATAGTGTCTCCTGAATGTCCTAT CC-3'
**F-ES$_1$**	5' - **TAMRA** - **CTTGAATTCCTG** - 3'
**F-ES$_2$**	5' - **FAM** - **CTTAAGCTTCTG** - 3'
**F-ES$_3$**	5' - **AMCA** - **CTTCTGCAGCTG** - 3'
**CS$_1$**	5' - **NH_2** - TTTTTTTTTTCAGGAATTCAAG - 3'
**CS$_2$**	5' - **NH_2** - TTTTTTTTTTCAGAAGCTTAAG - 3'
**CS$_3$**	5' - **NH_2** - TTTTTTTTTTCAGCTGCAGAAG - 3'

Supplementary Figures:

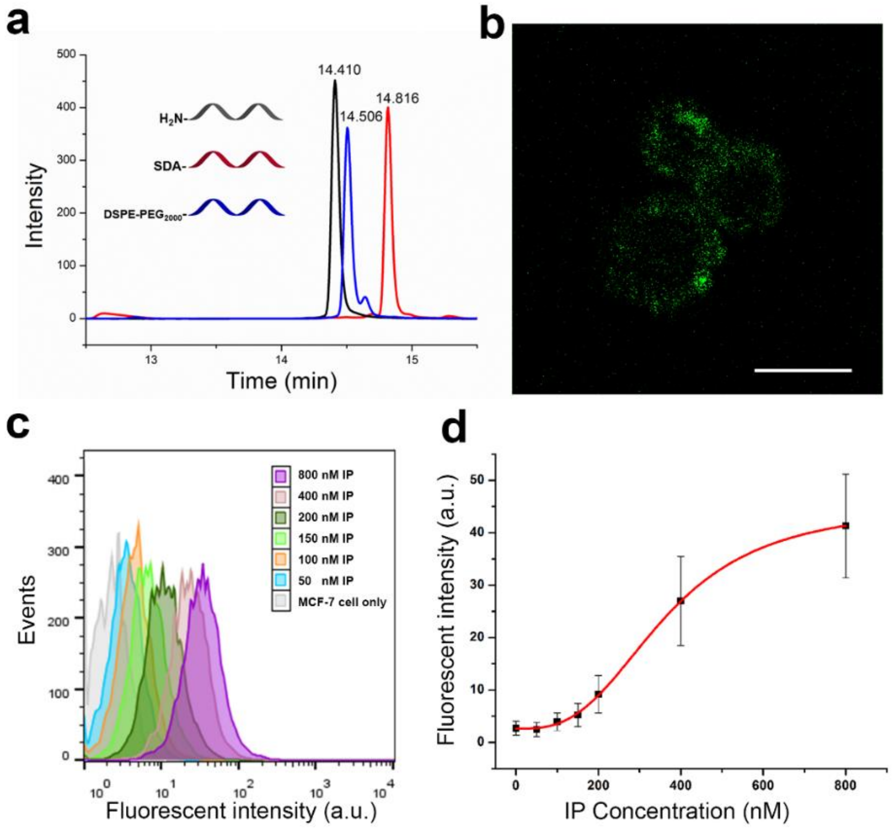

Supplementary Figure 1 Attachment of initiate primer (IP) to the cell surface. **a** HPLC characterization of the IP. IPs were chemically synthesized and functionalized with NH_2, SDA, and PEG_{2000}-DSPE at the 5′ ends, respectively. **b** Fluorescent observation of the anchored IPs on MCF-7 cells. The IP was labeled with FAM (green fluorescence). Scale bare, 20 μm. **c**, **d** respectively show the flow cytometry evaluation of the anchoring efficiency of IP and the corresponding calibration curve. Different concentrations of FAM-labeled IP (0, 50, 100, 150, 200, 400, 800 nM) were incubated with 2×10^6 MCF-7 cells at room temperature for 30 minutes, unbound IP was removed. Source data are provided as a Source Data file. The error bars in the calibration curve stand for the standard deviation from 10,000 cell events at each concentration.

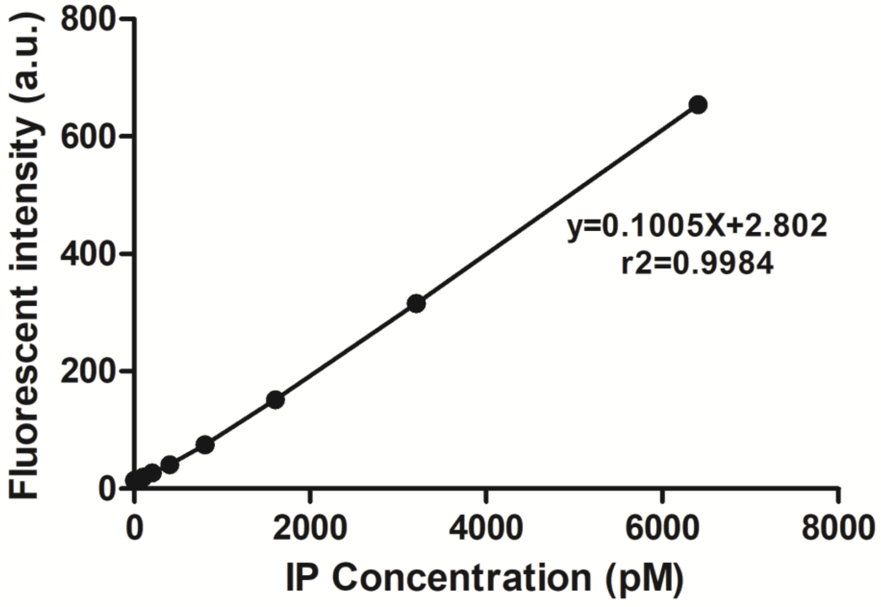

Supplementary Figure 2 Calibration curve of F-IP for the determination of cell-surface attached IP. Source data are provided as a Source Data file.

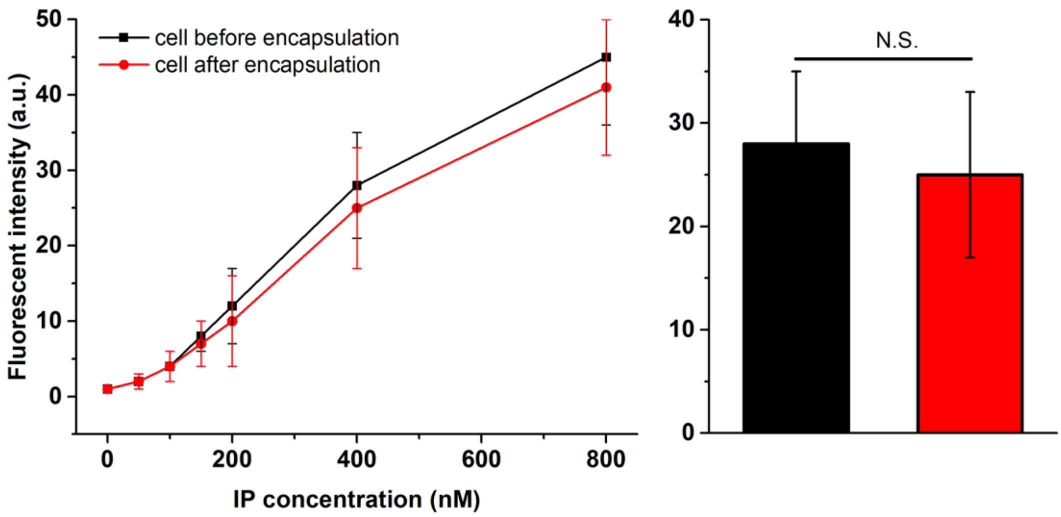

Supplementary Figure 3 Stability test of the surface attached IP. Fluorescent dye-labeled IP was used as the probe to monitor the stability, with different incubation concentrations of 0, 50, 100, 150, 200, 400, and 800 nM, respectively. The curves show the fluorescent intensities of cells before (black) and after (red) the encapsulation. The right histogram typically shows the average fluorescent intensity of the cells as a comparison before and after the encapsulation process when 400 nM IP is used for the experiment. Source data are provided as a Source Data file. Data are presented as of three independent experiments. Not Significant (N.S.) as determined by the one-way ANOVA test. The error bars indicate the standard deviation of 10,000 cell events at each concentration.

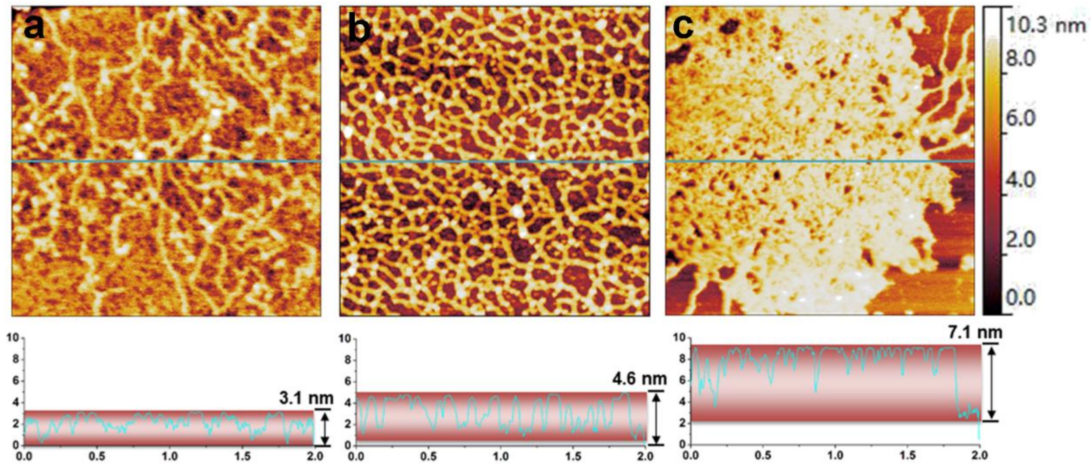

Supplementary Figure 4 AFM characterizations of the density of DNA cocoon. AFM height images of DNA networks on mica, scale 2 μm × 2 μm. isDOP were performed by using different concentrations of BP, **a** 20 nM, **b** 80 nM, and **c** 320 nM, respectively. Frequencies analysis showed the height changes of DNA polymers were 7, 13 and over 20 polymer strands per micrometer, indicating densities of DNA networks were increased. Increase thickness of the DNA networks was also observed.

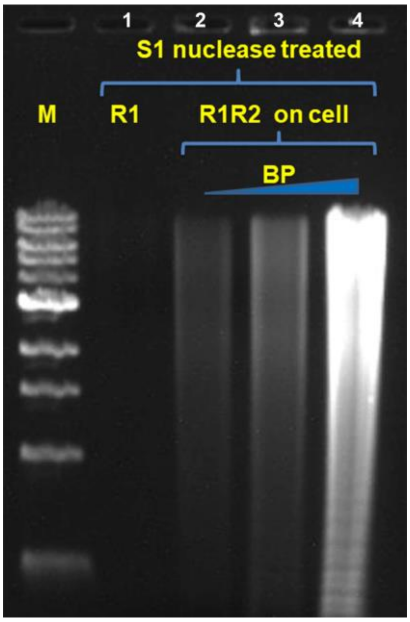

Supplementary Figure 5 S1 nuclease degradation test. To characterize the R1 and R2 reactions on cell, the degraded DNA products are analyzed by agarose gel analysis. Lane 1, solely conducted R1 on cell. Lanes 2-4, R1R2 reactions to assemble DNA network on cell. Increased concentrations of BP (5, 25, and 100 nM) are used to show the DNA polymer network formation by S1 nuclease degradation test. The DNA double-stranded structures are staining with GelRed. Source data are provided as a Source Data file.

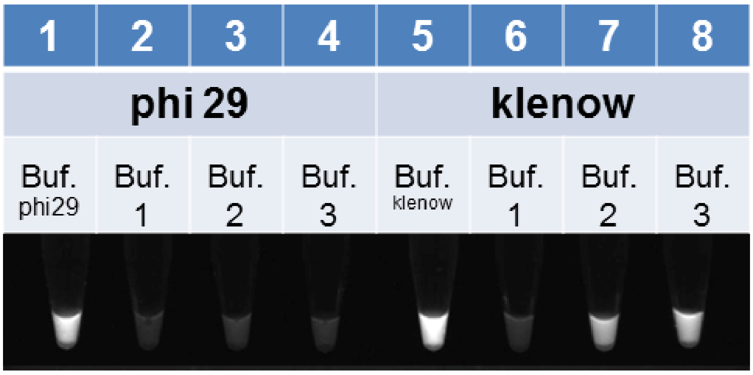

Supplementary Figure 6 Condition optimization for the polymerization reactions. Optimization of the reaction buffers and DNA polymerases used in isDOP. The details of buffers and enzymes are listed in Supplementary Table 1.

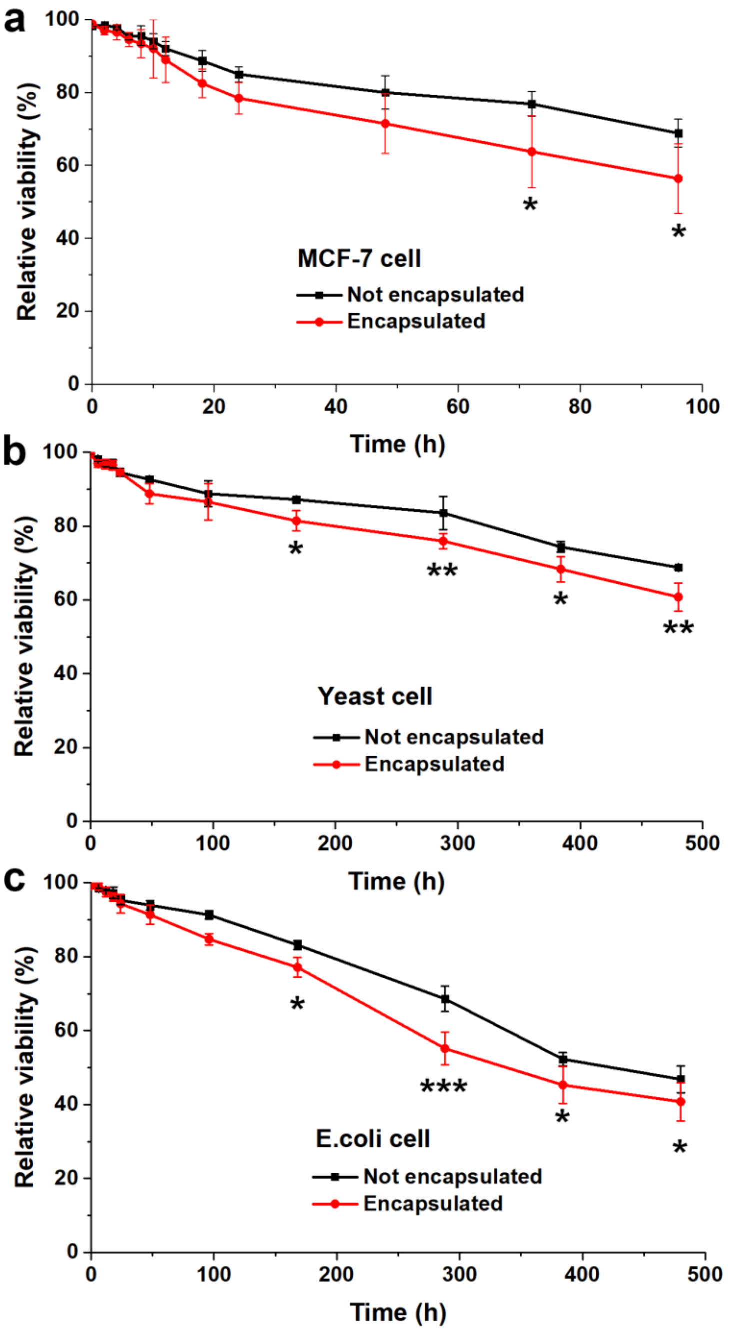

Supplementary Figure 7 Cell viability tests as a function of time. **a-c** respectively show the viabilities of MCF-7, yeast, and E.coli cells over time, the red curves. Cells without encapsulation were used as controls, the black curves. Source data are provided as a Source Data file. Data are presented as the mean ± s.d. *P<0.05. **P<0.01. ***P<0.001 *vs.* control group, as determined by the two-way ANOVA test. The error bars indicate the standard deviation of three replicates for each time intervals.

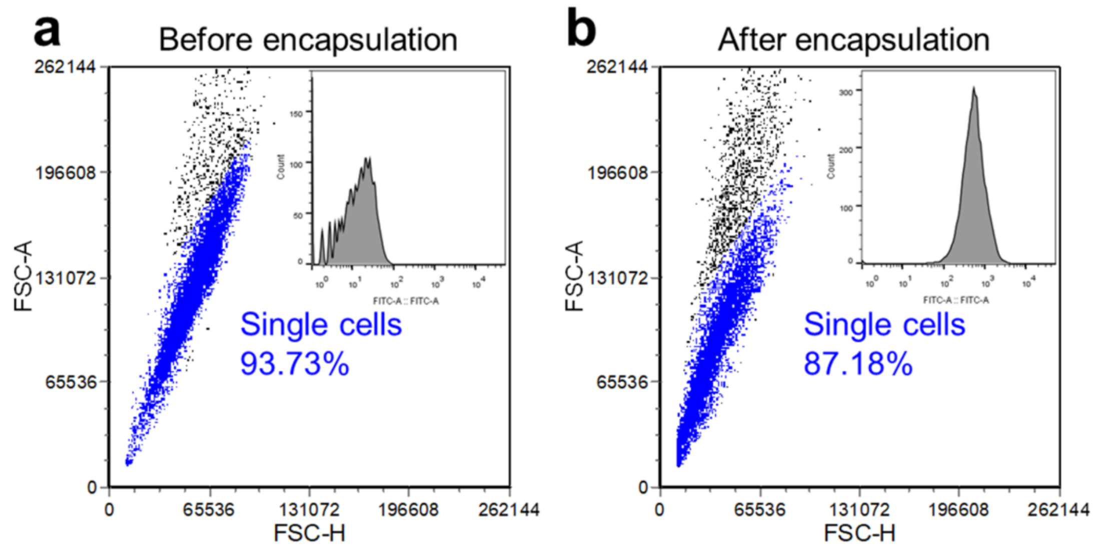

Supplementary Figure 8 Evaluation of single cell encapsulation ratio. Cytometry analysis by FSC-A vs. FSC-H plotting. The percentages show the single cell ratios, **a** before and, **b** after the encapsulation. Inserts are histograms of 10000 events.

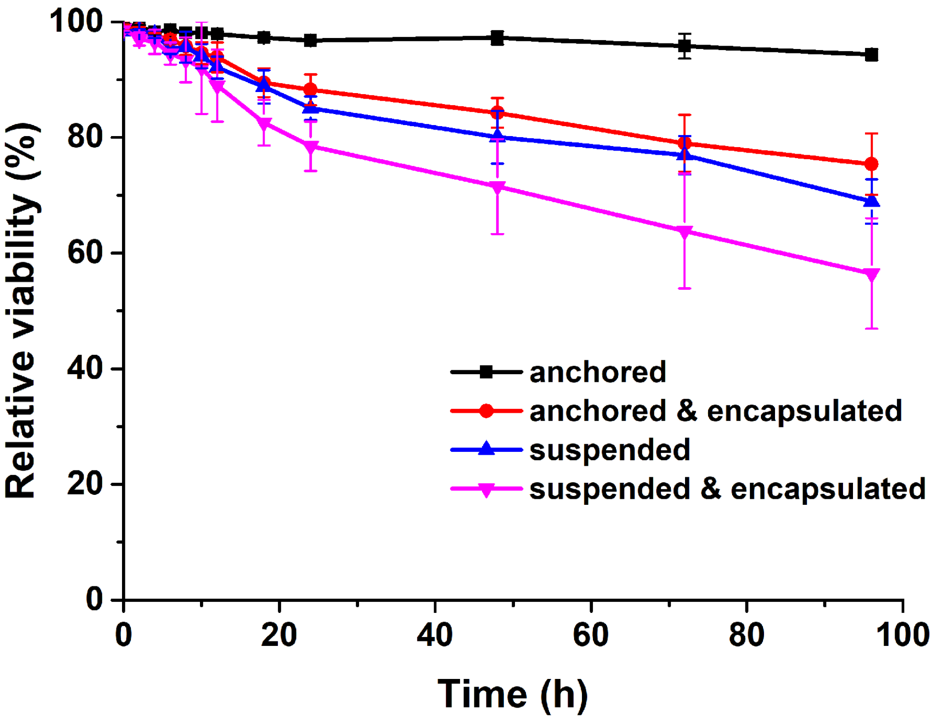

Supplementary Figure 9 Cell viability tests under different encapsulation conditions. Viability test of encapsulated MCF-7 cells as a function of time, showing the influence of different conditions on cell viabilities. The anchored and suspended cells without encapsulation are used as controls. Data are presented as the mean ± s.d. Source data are provided as a Source Data file. The error bars indicate the standard deviation of three replicates for each time intervals.

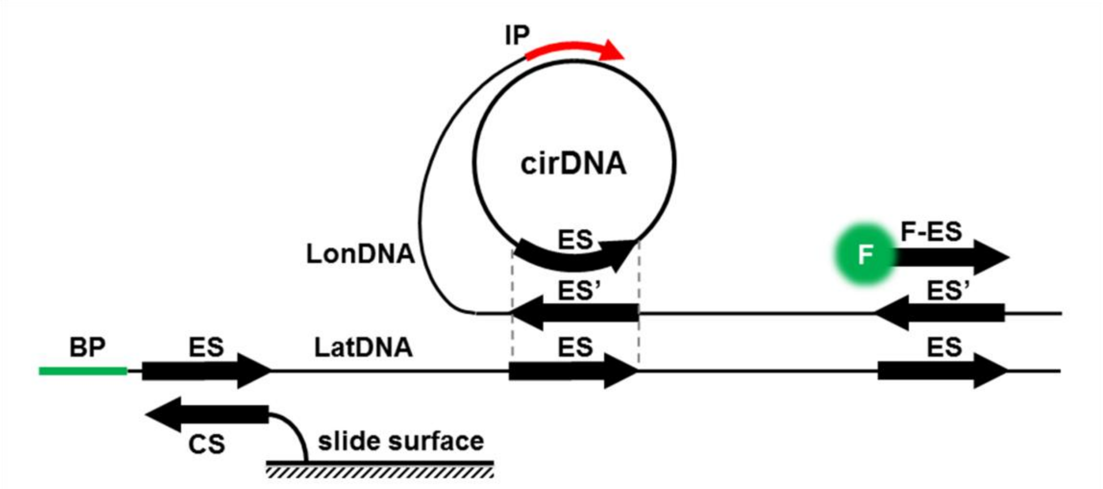

Supplementary Figure 10 Schematic illustration of the relationships of the DNA sequences designed in the experiment.

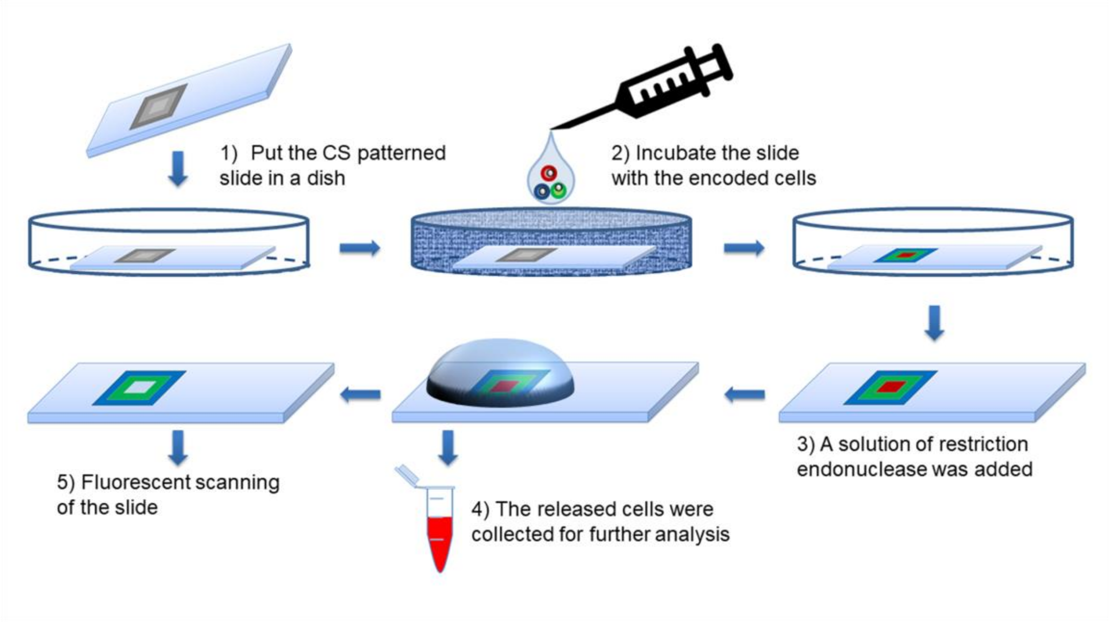

Supplementary Figure 11 Schematic illustration of the procedure for cell manipulation on the patterned slide surface.

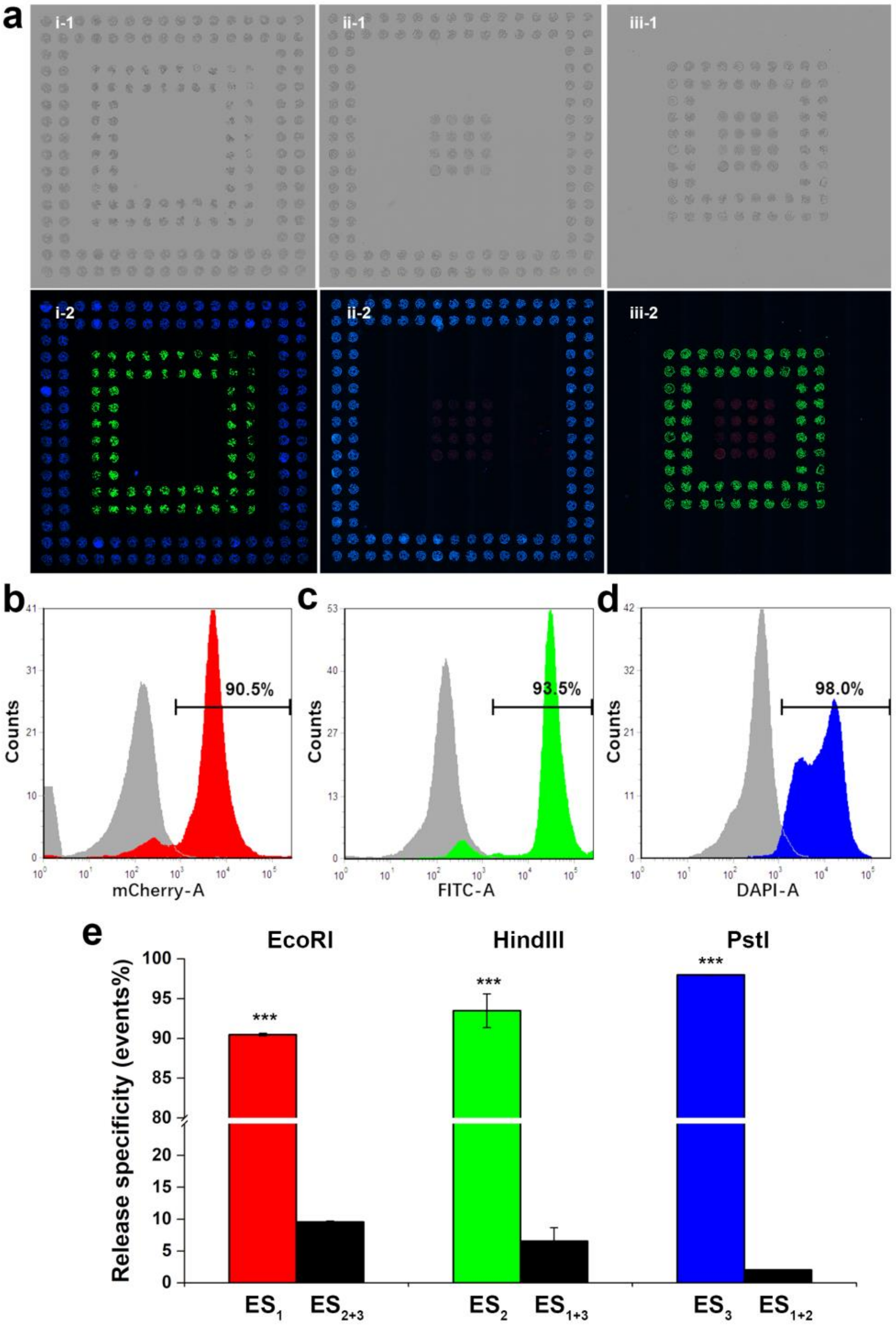

Supplementary Figure 12 Cell release efficiency and specificity. **a** Bright (top row) and fluorescent (bottom row) observations of the slides after treatments with 0.2 units μL^{-1} each of the EcoRI-HF, HindIII-HF, and PstI-HF. **b-d** The released ES_{1-3}-encoded cells in each treatment is recorded by flow cytometry analysis. 5000 events are recorded for each sample. **e** Histogram shows the release specificities for each of the ES_{1-3}-encoded cells. Data are presented as the mean ± s.d. ***P<0.001 *vs.* control group as determined by the one-way ANOVA test. Source data are provided as a Source Data file. The error bars indicate the standard deviation of three replicates.

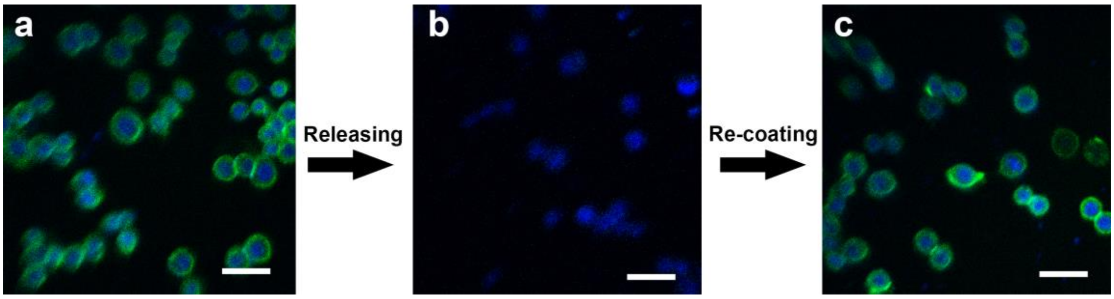

Supplementary Figure 13 Reversible release and coating of DNA cocoons on cells. **a** The MCF-7 cells attached in the culture dish are encapsulated with DNA cocoons. **b** After treatment with HindIII-HF, the surface DNA cocoons are digested. **c** Further incubation with the reaction system could encapsulate these MCF-7 cells again. The surface-grafted DNA cocoons are labeled with FAM-modified oligonucleotides (green). The cell nuclei were stained with Hoechst 33342 (blue). Scale bar, 50 μM.

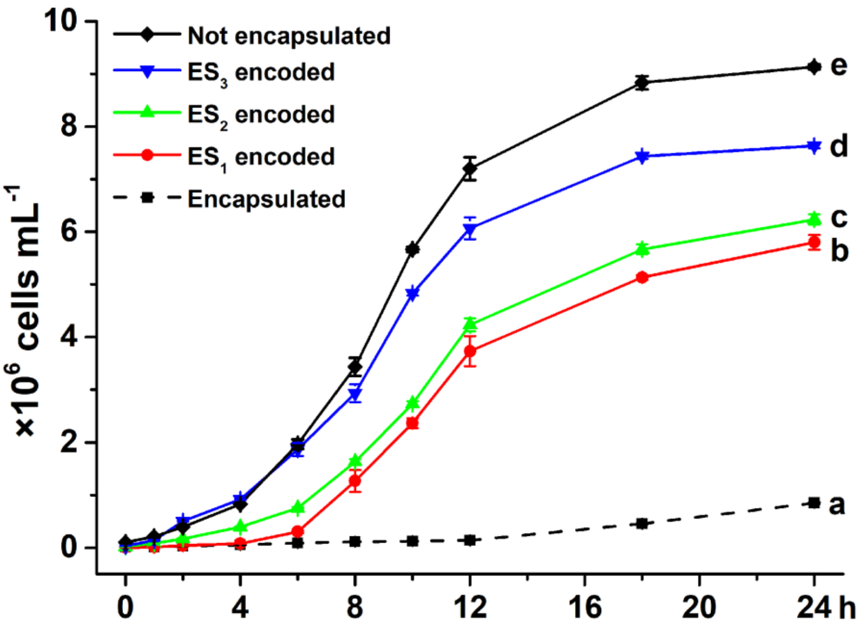

Supplementary Figure 14 Cell growth curves of the encapsulated cells. **a** before, and **b-d** after releasing from the ES_1, ES_2 and ES_3 encoded DNA cocoons, respectively. **e** Growth curve of the un-encapsulated cells. Cell viability data are presented as the mean ± s.d. Source data are provided as a Source Data file. The error bars indicate the standard deviation of three replicates for each time intervals.
