# Supplementary material for: Design and fabrication of flexible DNA polymer cocoons to encapsulate live cells
Source: Nat Commun. 2019 Jul 3;10:2946. doi: 10.1038/s41467-019-10845-2 (PMC6610073; doi:10.1038/s41467-019-10845-2)
Supplement: Supplementary file 3 — Description of Additional Supplementary Files [file 41467_2019_10845_MOESM3_ESM.pdf]

Description of Additional Supplementary Files

File name: Supplementary Movie 1

Description: 3D visualization of the DNA cocoon encapsulated cells. Confocal microscope scanning of the encapsulated MCF-7 cells was performed at a continue z-axis series. The acquired 2-D slice images were stacked together to make a movie over time.
